# The impact of social determinants of health on access to partial nephrectomy, minimally invasive surgery, and robotic-assisted approaches for renal cell carcinoma

**DOI:** 10.1007/s00345-026-06708-3

**Published:** 2026-08-18

**Authors:** Ronny Baqain, Laith Baqain, David Inkoo Lee, Sohrab Naushad Ali, Ryan W. Dobbs, Faris Najdawi, Mohammad Shahait

**Affiliations:** 1 University of Manchester, Manchester, UK; 2 Division of Gastroenterology and Hepatology, New York-Presbyterian Weill Cornell Medical Center, New York, NY USA; 3 UCI Health, Department of Urology, University of California, Irvine, CA USA; 4 Department of Urology, Cook County Health, Chicago, USA; 5 Department of Urology, University of California, Irvine, CA USA

**Keywords:** Renal cell carcinoma, Partial nephrectomy, Minimally invasive surgery, Robotic surgery, Social determinants of health, Health disparities

## Abstract

**Purpose:**

Social determinants of health (SDOH), including race/ethnicity, socioeconomic status (SES), insurance coverage, and geographic location, are established drivers of disparities in surgical access and outcomes. Robotic-assisted partial nephrectomy (RAPN) has become the preferred minimally invasive approach for localized renal cell carcinoma (RCC), yet the extent to which SDOH influence access to partial nephrectomy, minimally invasive surgery, and RAPN specifically, as well as postoperative outcomes has not been comprehensively characterized.

**Methods:**

A systematic review was conducted following PRISMA guidelines. PubMed/MEDLINE, Google Scholar, ScienceDirect, Embase, Web of Science, and Scopus were searched in October 2025 for studies evaluating associations between SDOH and surgical management or perioperative outcomes in patients with RCC. Fourteen studies met inclusion criteria. Methodological quality was assessed using the Newcastle-Ottawa Scale.

**Results:**

Fourteen retrospective observational studies comprising 918,452 patients were included. Lower socioeconomic status, non-White race/ethnicity, public or absent insurance, and treatment at low-volume or non-academic institutions were each independently associated with reduced receipt of guideline-concordant nephron-sparing surgery. Insurance status and race/ethnicity were the most consistently reported determinants. Socioeconomic disadvantage was also associated with increased postoperative complications, longer length of stay, and higher readmission rates.

**Conclusions:**

SDOH significantly influence both access to nephron-sparing and robotic-assisted surgery and perioperative outcomes in patients with RCC, independent of clinical factors. Because the included studies did not directly evaluate interventions, SDOH screening at referral, patient navigation, expansion of robotic capacity in safety-net hospitals, and reimbursement reform represent potential options for improving equitable delivery of minimally invasive nephron-sparing surgery.

## Introduction

Social determinants of health (SDOH) encompass the non-medical factors that shape an individual’s health outcomes, functional status, and access to care. These include race and ethnicity, socioeconomic status (SES), educational attainment, occupational environment, insurance coverage, geographic location, and broader societal structures and policies that govern resource distribution [1]. According to the World Health Organization, SDOH represent contextual drivers that contribute to population-level disparities in disease incidence, treatment access, and clinical outcomes [2]. These disparities are reinforced by a growing body of evidence across surgical care pathways [1, 3, 4].

Renal cell carcinoma (RCC) is a major global health burden and is among the most common malignancies of the genitourinary system [5]. For small renal masses, partial nephrectomy (PN) is the recommended standard of care because it provides oncologic control while preserving renal function [6]. Alternative management options include active surveillance and ablative therapies in select patients, as outlined in AUA guidance [7]. However, surgical resection remains the preferred approach for most clinically eligible patients [6–9]. Over the past decade, robotic-assisted partial nephrectomy (RAPN) has become the dominant minimally invasive approach because of its technical precision, reduced perioperative morbidity, and equivalent oncologic control [10].

Despite widespread adoption of the da Vinci robotic surgical system, disparities in access to RAPN persist [11–13]. Several SDOH have also been independently associated with increased postoperative complications, higher readmission rates, and prolonged length of stay (LOS), underscoring the importance of incorporating SDOH into perioperative care models [14].

The purpose of this review is to synthesize current evidence on the impact of SDOH on access to partial nephrectomy, minimally invasive surgery, and RAPN specifically, and on perioperative outcomes. Most administrative datasets and cancer registries do not record surgical modality at the level of robotic assistance, so much of the SDOH literature reports outcomes for partial nephrectomy (PN) or minimally invasive surgery (MIS) generally rather than RAPN specifically. Throughout this review, PN means nephron-sparing surgery by any approach (open, laparoscopic, or robotic), MIS means laparoscopic and robotic techniques together, and RAPN is used only where a study specifically evaluated the robotic-assisted technique. Where a study did not distinguish robotic technique, we describe its findings using its own terminology and treat them as relevant to, though not equivalent to, RAPN-specific access. By characterizing the role of social and institutional factors in shaping surgical care, this work aims to inform more equitable implementation of minimally invasive nephron-sparing surgery and guide future risk stratification models.

## Methods

### Search strategy and eligibility criteria

This systematic review was conducted in accordance with PRISMA guidelines. A comprehensive literature search was performed in October 2025 using PubMed/MEDLINE, Google Scholar, ScienceDirect, Embase, Web of Science, and Scopus. Search terms included “renal cell carcinoma,” “partial nephrectomy,” “robotic surgery,” “minimally invasive nephrectomy,” “social determinants of health,” “race,” “ethnicity,” “insurance,” “socioeconomic,” “education,” “income,” and “employment.” Studies were included if at least one SDOH variable was evaluated in relation to [1] utilization of partial nephrectomy versus radical nephrectomy [2], access to minimally invasive or robotic approaches, or [3] perioperative outcomes, including complications, readmissions, length of stay, or mortality.

### Study selection

**Fig. 1:**
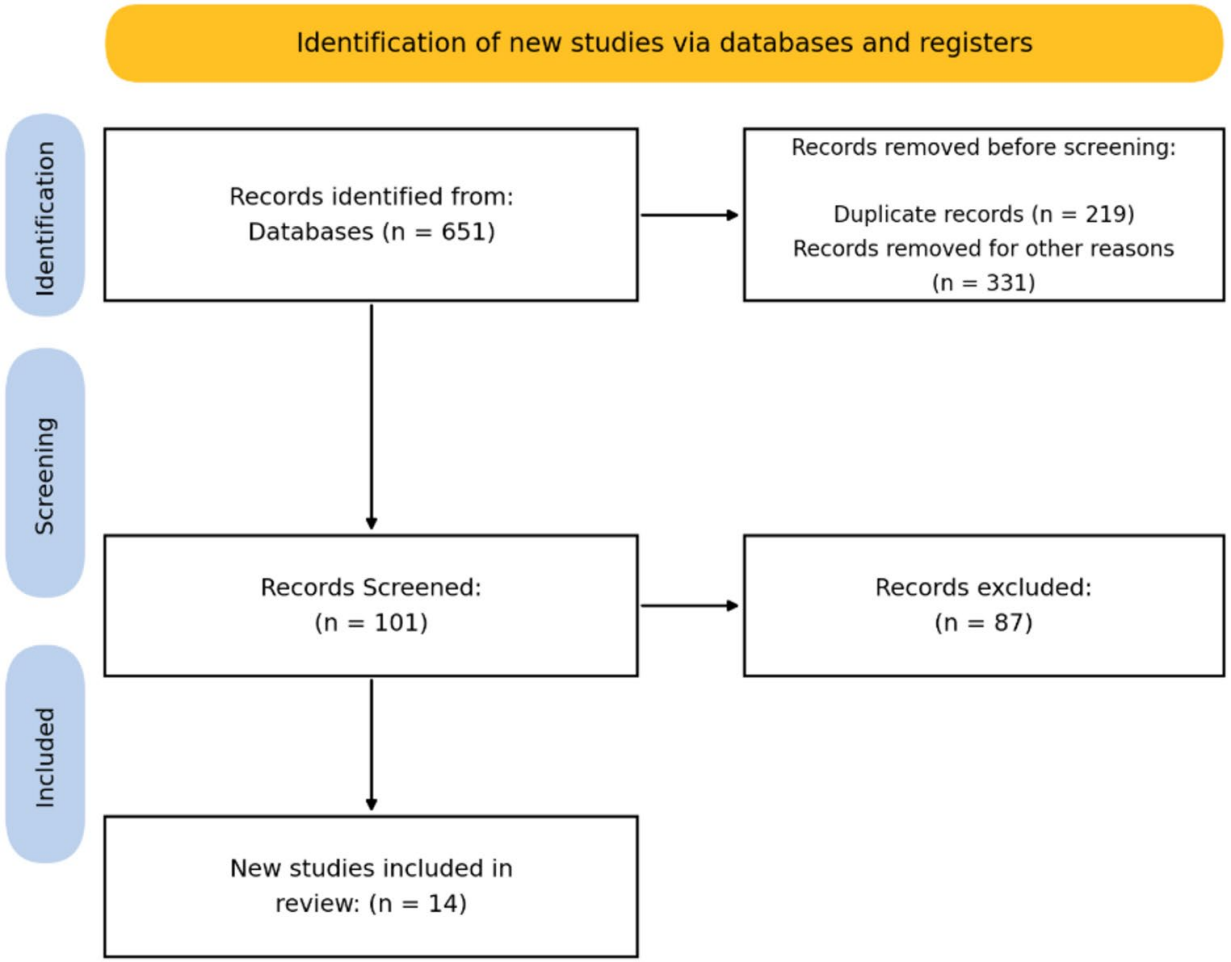
PRISMA flow diagram of study selection

Two reviewers (RB, LB) independently screened titles and abstracts to determine eligibility. Full-text articles were retrieved for studies that met inclusion criteria, and final eligibility was confirmed through detailed review. Disagreements were resolved by discussion, with adjudication by a senior reviewer (MS) when consensus could not be reached. The selection process is summarized in Fig. 1.

### Data extraction and quality assessment

**Fig. 2:**
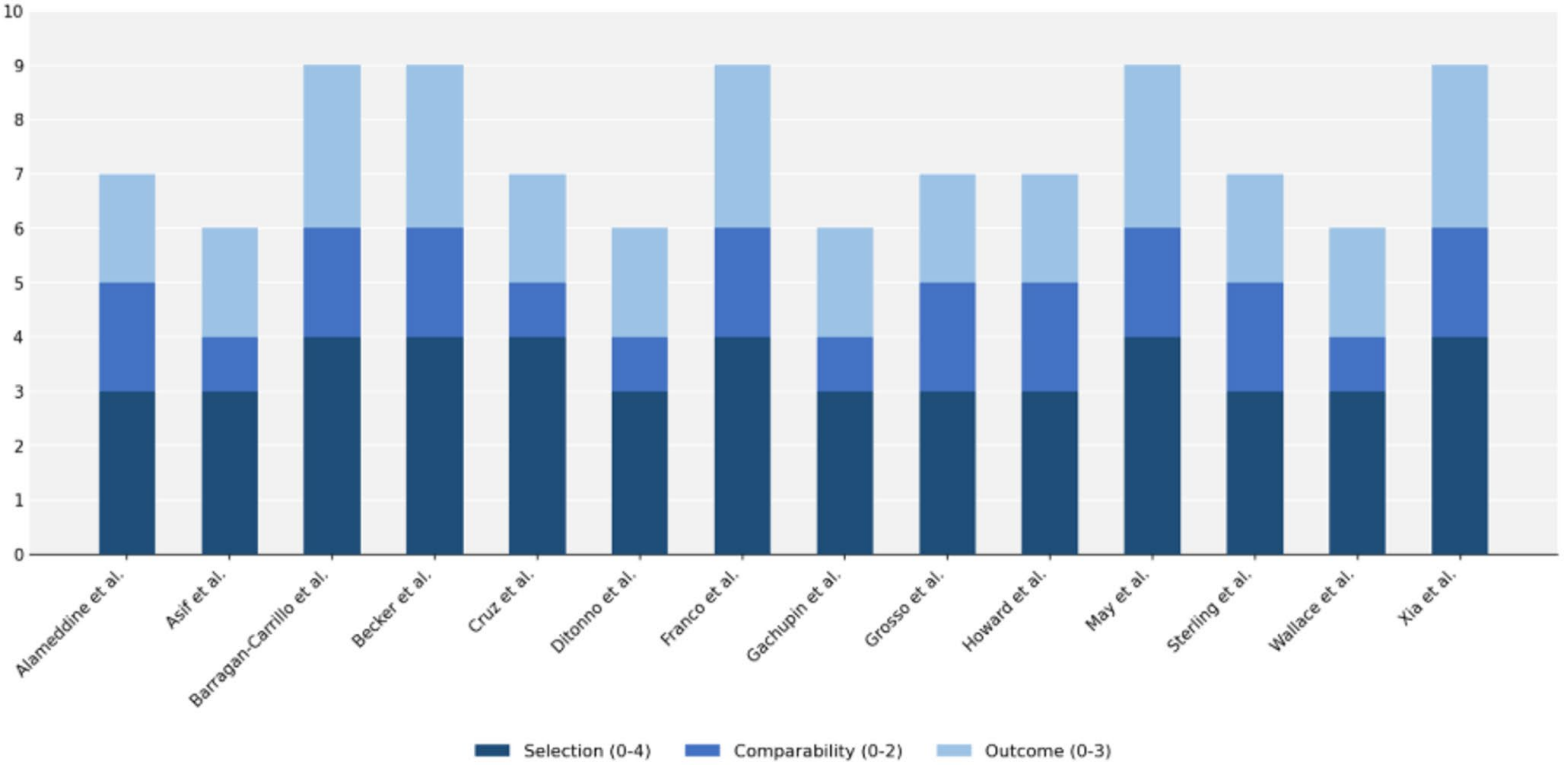
Methodological quality assessment of included studies using the Newcastle-Ottawa Scale (NOS)

Two reviewers (RB, LB) independently extracted data using a pre-specified form, including study design, patient population, SDOH domains, outcomes, and covariates in adjusted models. Disagreements were resolved through discussion, with third-reviewer adjudication when needed. Methodological quality was assessed independently using the Newcastle-Ottawa Scale (NOS), which evaluates selection, comparability, and outcome/exposure domains for a maximum score of 9. Ten of the 14 included studies were high quality (NOS > = 7), while four were moderate quality (score 6), mainly because of limited adjustment for confounders. Domain-level NOS scores are summarized in Fig. 2.

### Synthesis methods

Given the heterogeneity in study design, patient populations, SDOH variables examined, and outcome definitions, a quantitative meta-analysis was not performed. Instead, a narrative synthesis was conducted. Studies were grouped thematically by SDOH domain (race/ethnicity, insurance status, socioeconomic status, and geography) and by outcome category, including surgical approach, access to RAPN, and perioperative outcomes. Within each SDOH domain, findings are presented in the Results below in that order: studies addressing surgical approach or access to nephron-sparing and robotic-assisted surgery are described first, followed by studies addressing perioperative outcomes, so that access-related and outcome-related evidence remain distinguishable rather than pooled together.

## Results

### Study characteristics

This review included 14 studies published between 2013 and 2025. Thirteen studies were from the United States, and one was from Italy. All studies were retrospective observational studies and included a total of 918,452 patients with RCC. Study characteristics are summarized in Table 1.

**Table 1:** Characteristics of included studies

Study	SDOH	Outcomes	Date	Sample size	Country	Design
Alameddine et al. [15]	Race/Ethnicity, Insurance Status, Location	RAPN vs. Open PN	2019	23,154	USA	Retrospective Cohort Study
Asif et al. [16]	Race/Ethnicity, Social Deprivation Index	Perioperative Outcomes	2024	432	USA	Retrospective observational
Bararagan-Carrilo et al. [17]	Race/Ethnicity, Payor Status	PN vs. RN	2025	31,093	USA	Retrospective cohort study
Becker et al. [18]	Race/Ethnicity, Income, Education, Poverty Level, Region	Surgical vs. Non-Surgical Management, PN vs. RN	2013	26,468	USA	Retrospective Observational
Cruz et al. [19]	Race/Ethnicity, Neighborhood Characteristics	PN vs. RN	2022	238,141	USA	Retrospective Cohort Study
Ditonno et al. [20]	Insurance Status, Region	Renal Ablation vs. MIPN vs. MIRN, LOS	2024	80,821	USA	Retrospective observational cohort
Franco et al. [21]	Region, Insurance Status	Perioperative Outcomes, LOS, Readmission Open PN, vs. MIPN	2024	65,325	USA	Retrospective Cohort study
Gachupin et al. [22]	Race/Ethnicity, Neighborhood Socioeconomic Factors	No Treatment vs. Nephrectomy, PN vs. RN	2022	5,111	USA	Retrospective Cohort Study
Grosso et al. [23]	Income Level, Education	PN vs. RN, Peri and Postoperative Outcomes	2024	1,042	Italy	Retrospective Cohort Study
Howard et al. [24]	Race/Ethnicity, Insurance	Non-Guideline Treatment (Surveillance or Nephron-Sparing Surgery)	2021	158,445	USA	Retrospective Cohort Study
May et al. [25]	Income, Race/Ethnicity, Insurance	PN vs. RN, LOS, Mortality, Follow up	2019	99,035	USA	Retrospective Cohort Study
Sterling et al. [26]	Income, Insurance, Race/Ethnicity, Distance to Facility, Education	Use of PN or Minimally Invasive Surgery	2020	69,694	USA	Retrospective Cohort
Wallace et al. [27]	Race/Ethnicity, Socioeconomic Status	Use of PN	2022	5,633	USA	Retrospective cohort
Xia et al. [28]	Race/Ethnicity, Insurance, Socioeconomic Status	PN vs. RN	2019	84,058	USA	Retrospective Cohort study

### Socioeconomic status and income

Seven studies evaluated the association between SES, income level, or area-level deprivation and management of renal masses; three also examined perioperative or postoperative outcomes [15–21]. Several studies demonstrated that lower SES was associated with a reduced likelihood of receiving partial nephrectomy, whether open or robotic, and with lower utilization of minimally invasive surgery, with a corresponding increase in radical nephrectomy use [16, 18–21]. Similarly, lower household income, defined as less than $62,000 per year, was independently associated with a reduced likelihood of receiving minimally invasive or nephron-sparing surgery [19]. Regarding perioperative outcomes, area-level deprivation, assessed using the Social Deprivation Index, was also associated with greater perioperative risk, amplifying the relationship between obesity and longer ischemia time and higher estimated blood loss [15]. Socioeconomic disadvantage was further associated with increased postoperative complications, longer LOS, and higher 30-day readmission rates [19, 22, 23].

### Race and ethnicity

Eleven studies examined race and ethnicity as determinants of surgical management for renal masses, including use of partial nephrectomy, radical nephrectomy, minimally invasive surgery, and guideline-concordant care [24–27], 28– [21]. Across multiple cohorts, non-Hispanic Black, Hispanic, American Indian/Alaska Native, and selected Asian patient groups were less likely to receive partial nephrectomy or minimally invasive surgery and were more likely to undergo radical nephrectomy than non-Hispanic White patients. These findings persisted after adjustment for tumor characteristics, comorbidities, and insurance status [16, 24, 25], 28– [21]. Subgroup analyses demonstrated heterogeneity within racial and ethnic categories. For example, in Hispanic populations, Mexican/Chicano patients had the highest likelihood of undergoing radical nephrectomy, whereas South/Central American patients were comparatively less likely to undergo radical nephrectomy [26]. When controlling for healthcare access and neighborhood-level socioeconomic factors, race-based differences in the likelihood of undergoing partial nephrectomy were attenuated but remained after adjustment, suggesting an independent effect beyond measured socioeconomic factors. A separate registry study found that American Indian/Alaska Native patients were more likely to undergo radical rather than partial nephrectomy compared with non-Hispanic White patients, and that Mexican American patients had higher odds of receiving no surgical treatment at all; both associations weakened but did not disappear after adjusting for neighborhood-level socioeconomic factors [27].

Race and ethnicity also influenced perioperative risk. Obesity-associated increases in ischemia time and estimated blood loss were more pronounced in non-Hispanic White patients and in those living in high-deprivation neighborhoods. Specifically, body mass index > = 35 kg/m2 was associated with more than five-fold higher odds of ischemia time exceeding 18.5 min, and class I-III obesity conferred more than two-fold higher odds of estimated blood loss greater than 150 mL in high-deprivation settings [15]. However, disparities were no longer observed among American Indian/Alaska Native patients residing in higher-income, higher-education neighborhoods [26].

### Insurance status

Seven studies assessed insurance or payor status as a determinant of surgical management, including nephrectomy type, minimally invasive approach, and guideline-concordant treatment [22, 24, 25], 28– [19, 21]. Across large population-based cohorts, patients with Medicare, Medicaid, or no insurance coverage were less likely to undergo partial nephrectomy or minimally invasive surgery and were more likely to receive radical nephrectomy than privately insured patients. These findings persisted after adjustment for tumor characteristics and comorbidity burden [18, 19, 21, 24]. Insurance status was also associated with increased receipt of non-guideline-based treatment for kidney cancer [28]. More recent analyses confirmed that payor status remained a significant determinant of nephrectomy type and of robotic or laparoscopic utilization after adjustment for race and clinical factors [22, 23, 25]. Perioperative outcomes specifically by insurance status were not separately reported in the included studies.

## Discussion

This review demonstrates that SDOH exert a significant impact on access to RAPN and perioperative outcomes in patients with RCC. Importantly, these disparities persist despite adjustment for tumor characteristics and patient-specific comorbidities, indicating a substantial influence of systemic and social factors. The findings highlight that advances in surgical technology alone are insufficient to ensure equitable care and may, in some settings, exacerbate existing inequities in healthcare.

The association between lower SES and reduced likelihood of undergoing RAPN likely reflects several interacting mechanisms. Lower SES is associated with reduced referral to high-volume centers where RAPN is routinely performed. Limitations in transportation, financial constraints, and reduced healthcare literacy contribute to delayed diagnosis and lower availability of nephron-sparing surgery [29–31]. These barriers may also bias care toward more readily available or logistically simpler approaches, such as radical nephrectomy, leading to unfavorable patient outcomes [16, 18, 19].

RAPN requires specialized infrastructure, experienced surgical teams, and institutional investment, which are more commonly concentrated in tertiary or academic centers [32]. Patients with fewer financial and social resources face barriers to accessing these centers, including travel costs, time away from work, and caregiving responsibilities. As a result, SES influences where patients receive care, which treatment options are available, and the likelihood of receiving minimally invasive nephron-sparing surgery [33].

This review also found a clear association between race/ethnicity and the choice of surgical intervention. These differences persisted after adjustment for tumor characteristics, comorbidities, and insurance status, highlighting the role of systemic bias in surgical care [24–26, 28]. Among American Indian/Alaska Native patients living in higher-income, higher-education neighborhoods, disparities in the likelihood of undergoing partial nephrectomy were no longer observed, suggesting that socioeconomic context may mediate some race-associated differences in this population.

Racial and ethnic minority patients are more likely to experience delayed diagnosis and interruptions in care because of barriers in primary care access, specialist referral, and insurance stability [34, 35]. These factors may result in presentation at more advanced stages or with greater medical complexity, indirectly limiting eligibility for minimally invasive surgery [21, 24, 28]. Such disparities reflect differences in healthcare access rather than intrinsic biological variation, underscoring the need for coordinated approaches that improve access and continuity of kidney cancer care.

Insurance status represents one of the most prominent social determinants of access to RAPN. Publicly insured or uninsured patients are consistently less likely to receive guideline-concordant minimally invasive surgery, even when clinically eligible [22, 24, 25, 28]. This is likely related, at least in part, to restricted referral networks, longer wait times, and limited availability of robotic platforms in safety-net institutions [36].

Insurance-related disparities also highlight how reimbursement structures shape institutional behavior. High up-front costs associated with robotic surgery may disincentivize its use for patients with lower reimbursement rates, reinforcing a two-tiered system of surgical care [37]. Beyond surgical selection, insurance status affects perioperative care in multiple ways. Patients with public or unstable insurance coverage may experience delays in preoperative workup, reduced access to care coordination, and less timely follow-up after discharge, all of which can affect readiness for surgery and postoperative recovery [38].

Beyond surgical selection, SDOH influence postoperative recovery and complication risk. Socioeconomic disadvantage may limit access to prehabilitation, nutrition optimization, and postoperative support, thereby increasing vulnerability to complications and prolonged hospitalization [30, 31]. Transportation barriers, unstable housing, and limited caregiver support can also delay discharge and increase readmission risk [20].

These dynamics have important implications as RAPN programs increasingly adopt enhanced recovery after surgery protocols and same-day discharge strategies [39]. While these models improve efficiency and patient experience for some, they may disproportionately benefit patients with stable housing, reliable transportation, and strong social support [40]. Without deliberate incorporation of SDOH into patient selection and discharge planning, such innovations risk widening disparities.

### Broader implications

The patterns identified in this review are consistent with what has been reported across other surgical specialties, where SDOH have repeatedly been shown to shape access to advanced and minimally invasive procedures independently of clinical factors [1, 2]. In the context of RAPN, this is especially consequential given the pace at which robotic surgery is being adopted as a standard of care for localized RCC. As uptake increases, there is a risk that the benefits will accrue predominantly to patients who are already well positioned to access specialist care, while those facing socioeconomic disadvantage, insurance barriers, or geographic distance from high-volume centers are directed toward less optimal alternatives [12]. Concentrating robotic capacity in tertiary academic settings without parallel investment in safety-net and community hospitals risks building inequity into the structure of care delivery. Expanding robotic capacity in under-resourced settings also means real costs in equipment, maintenance, and surgeon training [41]. None of the included studies looked at whether this expansion is cost-effective or how it should be prioritized, and that gap needs to be addressed before capacity expansion is recommended as policy. Beyond access to robotic platforms themselves, disparities may also arise from unequal availability of adjunct planning technologies, such as three-dimensional virtual models and augmented-reality navigation, a concern recently raised in the RAPN literature [42]. At the level of clinical practice, referral patterns deserve particular attention, as socioeconomically disadvantaged and minority patients are less likely to reach centers where RAPN is routinely performed, and less likely to complete the pathway even when referred [30]. Reimbursement structures that disincentivize minimally invasive surgery for publicly insured patients may compound this further [12]. None of the included studies directly tested these or other interventions; Structured SDOH screening at referral, patient navigation, and insurance reform advocacy represent potential options for closing these gaps, though their real-world impact remains unknown.

International applicability of these findings warrants caution, as 13 of the 14 included studies originated from the United States, where insurance status and payor structure are closely tied to socioeconomic status. Only one included study was conducted in a universal healthcare system: an Italian cohort in which income and education level remained independently associated with surgical approach and perioperative outcomes despite universal insurance coverage [17]. This suggests that socioeconomic disparities in access to nephron-sparing and robotic-assisted surgery can persist even without insurance-based barriers, but one study cannot tell us how far this extends to other universal-coverage systems, which likely differ from the US in referral patterns and resource allocation. More work outside the US is needed to know how widely these disparities apply and what drives them.

### Limitations

All included studies were retrospective, and most relied on administrative databases and cancer registries that were not designed to capture SDOH. Race, SES, and insurance status were operationalized inconsistently across studies, limiting comparability and precluding formal meta-analysis. The literature was also heavily concentrated in the United States, and it remains uncertain how transferable these findings are to health systems with universal coverage or different patterns of surgical resource distribution. Most studies identified disparities without examining the mechanisms underlying them. Knowing that uninsured patients are less likely to receive RAPN is informative, but understanding where and why this occurs along the referral and treatment pathway is necessary to design effective interventions. Publication bias toward positive findings in this area is also plausible.

## Conclusions

SDOH consistently influence access to nephron-sparing and robotic-assisted surgery and perioperative outcomes in patients with RCC, independently of clinical factors. Lower SES, non-White race/ethnicity, public or absent insurance, and treatment at low-volume institutions each reduce the likelihood of receiving guideline-concordant nephron-sparing surgery. None of the included studies directly tested interventions to close these gaps; SDOH screening at referral, patient navigation, and investment in robotic capacity at safety-net hospitals represent potential options, though their cost-effectiveness and resource allocation impact remain unknown. Future prospective work should evaluate whether such strategies can translate documented disparities into measurable improvements in equity of access.

## Data Availability

No datasets were generated or analysed during the current study.
